# Supplementary material for: Stacking Model‐Based Classifiers for Dealing With Multiple Sets of Noisy Labels
Source: Biom J. 2025 Mar 12;67(2):e70042. doi: 10.1002/bimj.70042 (PMC11898607; doi:10.1002/bimj.70042)
Supplement: Supplementary file 1 — Supporting Information [file BIMJ-67-e70042-s001.zip › density-based-ensemble-model-main/competitors_real_data/netlab-master/netlab-master/help/gmmpost.htm]

Netlab Reference Manual gmmpost


# gmmpost

## Purpose

Computes the class posterior probabilities of a Gaussian mixture model.

## Synopsis

```
function post = gmmpost(mix, x)
```

## Description

This function computes the posteriors `post` (i.e. the probability of each
component conditioned on the data `p(j|x)`) for a Gaussian mixture model.
The data structure `mix` defines the mixture model, while the matrix
`x` contains the data vectors. Each row of `x` represents a single
vector.

## See Also

`gmm`, `gmmactiv`, `gmmprob`

---

**Pages:**
Index


---

Copyright (c) Ian T Nabney (1996-9)
